# Magnetic Resonance Enterography and Capsule Endoscopy in Patients Undergoing Patency Capsule for the Evaluation of Small Bowel Crohn's Disease: A Korean Clinical Experience

**DOI:** 10.1155/2020/8129525

**Published:** 2020-04-04

**Authors:** Hyun Seok Lee, Yun Jeong Lim, Jin-Hee Jung, Ji Hyung Nam, Junseok Park, Sun Hyung Kang, Ki Bae Kim, Hoon Jai Chun

**Affiliations:** ^1^Department of Internal Medicine, School of Medicine, Kyungpook National University, Kyungpook National University Hospital, Daegu, Republic of Korea; ^2^Department of Internal Medicine, Dongguk University College of Medicine, Dongguk University Ilsan Hospital, Goyang, Republic of Korea; ^3^Department of Radiology, Dongguk University College of Medicine, Goyang, Republic of Korea; ^4^Department of Internal Medicine, Soonchunhyang University College of Medicine, Seoul, Republic of Korea; ^5^Department of Internal Medicine, Chungnam National University School of Medicine, Daejeon, Republic of Korea; ^6^Department of Internal Medicine, Chungbuk National University College of Medicine, Chungbuk National University Hospital, Cheongju, Republic of Korea; ^7^Department of Internal Medicine, Korea University College of Medicine, Seoul, Republic of Korea

## Abstract

**Objective:**

Studies comparing magnetic resonance enterography (MRE) and capsule endoscopy (CE) for the assessment of small bowel (SB) Crohn's disease (CD) are scarce in Korea. In addition, there is no Korean experience of patency capsule (PC) examination prior to CE. The primary aim of this study was to compare diagnostic yields of MRE and CE for the assessment of SB CD. Secondary objectives were to compare the detection rate of proximal SB lesions by each modality in the Montreal classification and evaluate the safety and feasibility of PC in Korean CD patients.

**Methods:**

MRE was performed as the first examination to assess SB CD. PC examination and CE were then performed. Diagnostic yields of active SB disease by MRE and CE were then analyzed.

**Results:**

Disintegration of the patency capsule was shown in 5 patients out of 26 patients, who did not undergo CE. These 5 patients were accounted as negative CE findings. Overall, MRE and CE detected 80.8% and 65.4% of active SB lesions of CD in 26 patients, respectively (*P* = 0.212). MRE and CE detected 0% (0/26) and 19.2% (5/26) (*P* = 0.051) of jejunal lesions, 30.8% (8/26) and 42.3% (11/26) (*P* = 0.388) of proximal ileal lesions, and 80.8% (21/26) and 53.8% (14/26) (*P* = 0.039) of terminal ileal lesions, respectively. According to the Montreal classification, MRE and CE independently detected proximal disease (L4) in 30.8% (8/26) and 53.8% (14/26) (*P* = 0.092), respectively.

**Conclusions:**

The diagnostic yields of MRE and CE for the assessment of SB CD including proximal SB lesions were similar. MRE is a more objective tool for detecting clinically relevant stricture than PC although PC examination could be performed safely before CE to prove the patency of SB. This trial is registered with KCT0004305.

## 1. Introduction

Crohn's disease (CD) is a chronic relapsing inflammatory disease with mucosal and transmural inflammation of the bowel wall. The small bowel (SB) is a commonly affected lesion. CD is limited to the SB in 30% of patients. Involved SB lesions are frequently proximal to the terminal ileum. Thus, visualization of most parts of the small bowel is not possible with conventional ileocolonoscopic evaluation [1–3].

SB capsule endoscopy (CE) allows direct visualization of the entire SB in a minimally invasive procedure. It is commonly used for the evaluation of CD. The most common and serious adverse event of CE is retention due to small bowel stricture. Retention could lead to surgical intervention to remove the capsule [4, 5].

The PillCam patency capsule (PC) is a soluble capsule that consists of lactose and barium. If the PC is retained in the SB, it starts to dissolve at 30 hours after ingestion. PC examination could evaluate the patency of the SB before giving an actual CE. PC is useful for reducing the risk of capsule retention and expanding the application of CE to more cases of SB CD [1, 6–8].

By contrast, computed tomographic enterography (CTE) and magnetic resonance enterography (MRE) are radiological noninvasive techniques to evaluate CD and assess both intestinal and extraintestinal disease [9, 10]. MRE does not use ionizing radiation. It has better soft tissue contrast resolution compared to CTE [11, 12]. Each modality such as MRE and CE has its own strength and weakness. The most effective approach for assessing SB CD has not been definitely established [13].

In recent years, MRE has been introduced as an important tool for the assessment of CD in Korea [14]. However, there is no previous Korean study on the comparison of MRE and CE for the assessment of SB CE. Furthermore, PC has been very rarely used in Korea because PC examination could not be covered by the Korean national medical insurance program. There is no previous study on the experience of PC examination prior to CE.

Thus, the primary aim of this study was to compare diagnostic yields of MRE and CE for the assessment of SB CD. Secondary objectives were to compare the detection rate of proximal SB lesions by each modality in the Montreal classification and evaluate the safety and feasibility of PC in Korean CD patients.

## 2. Materials and Methods

### 2.1. Patients

This multicenter study recruited patients with established SB CD between May 2019 and October 2019. Patients aged greater than 18 years and those with established SB CD were included. Exclusion criteria were (1) constipation (patients with fewer than two bowel movements per week), (2) ileus, and (3) severe comorbidities.

Patients underwent MRE as the first examination to assess the SB CD and rule out strictures. PC examination was then performed for all patients. CE examination was subsequently performed. MRE and CE were performed within four weeks. The protocol was registered with the Clinical Research Information Service (a representative clinical trials registry platform in Korea, registration number: KCT0004305). The study was performed in accordance with the guidelines of the Declaration of Helsinki. It was approved by the Institutional Review Board (2019-03-D12).

### 2.2. Magnetic Resonance Enterography

All examinations were performed using a 3.0-T MR unit (Skyra; Siemens Medical Solutions, Erlangen, Germany). One liter of polyethylene glycol (PEG) water solution (Coolprep powder, Taejoon, Korea) was administered 40 minutes before MRE. It was used as an intraluminal contrast agent. Intravenous scopolamine butylbromide 30 mg/mL (Hyspan Inj, Huons, Korea) was given iv to reduce peristalsis and to prolong SB distension. When the distention quality in the jejunum and proximal ileum was unsatisfactory, images were obtained 30 minutes after the ingestion of a further 500 mL of PEG water solution. MR protocol included T2-weighted sequences in the axial and coronal planes and T1-weighted sequences in the coronal plane before and after gadoterate meglumine injection (Unilay at a dose of 0.2 mL/kg body weight and injection rate of 2 mL/sec). The section thickness was 5 mm with T2-weighted sequences in the axial and coronal planes and 3 mm with T2-weighted sequences in the coronal plane.

Patients with SB wall thickening (>3 mm), hyperenhancement, mural edema, comb sign (a sign of engorged vasa recta), enlarged lymph nodes, or the presence of ulcers were considered having signs of SB CD by MRE criteria [15, 16]. Strictures were defined as a change in a bowel caliber with dilatation of the proximal segment above 2.5 cm [1, 17]. MRE interpretations were assessed by two radiologists with more than 5 years of experience in the evaluation of IBD lesions who were blind to the results of CE.

There is a lack of standardized accepted division of the SB on MRE. The following segmentation was used to define each SB segment. The terminal ileum was considered the distal 15 cm proximal to the ileocecal valve or ileocolonic junction. The proximal ileum was considered the SB located on the left lower inferior quadrant and upper right quadrant, whereas the jejunum was considered the SB located on the left quadrant [15, 18].

### 2.3. Patency Capsule

Patients received a PC (Medtronic, Minneapolis, MN, USA) right after or within a maximal interval of two weeks after undergoing MRE. If PC examination was not performed on the same day of MRE, patients were restricted to a liquid low-fiber diet for one day with an overnight fast prior to PC examination.

Patients were asked to trace their stool to detect excretion of the PC. In cases where the PC was not found to be excreted intact, its location was verified on a plain abdominal film at 30 hours after ingestion. If the PC was not observed on an abdominal X-ray image, this was defined as a complete PC passage.

### 2.4. Capsule Endoscopy

When PC examination showed patency of SB, actual CE examinations were performed for patients at a maximal interval of four weeks after undergoing MRE. A PillCam (SB3; Medtronic, Minneapolis, MN, USA) or a MiroCam (MC4000; IntroMedic, Seoul, South Korea) was used for CE examinations. To improve visualization of the SB, patients were given 2 L of PEG solution two hours before capsule ingestion.

Capsule retention (CR) was defined as capsule remaining in the GI tract for at least two weeks after ingestion with retention confirmed by abdominal radiography or when endoscopic or surgical interventions were required to remove the capsule [5, 19, 20].

Images of CE were analyzed using the RAPID 8.1 version software (Medtronic) or the MiroView 4.0 version software (IntroMedic). CE was assessed by three gastroenterologists with more than 10 years of experience in gastrointestinal endoscopy and a minimum of 50 previous CE readings who were blinded to the results of MRE. SB was divided into three segments: the jejunum, proximal ileum, and terminal ileum using 2–4 last minutes of images before CE reached the cecum and corresponding approximately to the distal 15 cm of terminal ileum [15]. Images of CE were judged as negative (or inactive) if no abnormalities were found. They were considered positive (or active) if more than three SB ulcerations (aphthous lesions or ulcers) were observed [17, 21].

The quality of the CE image was evaluated according to the proportion of the SB mucosa visualized without debris, liquid, or bubbles. It was categorized as excellent (≥75% visualization of the SB mucosa was achieved), good (50%-74% of the mucosa was in perfect condition), fair (25%-49% of the mucosa was under perfect conditions), or poor (≤24% of the mucosa could be observed) [22].

### 2.5. Proximal Small Bowel Lesion in the Montreal Classification

The proximal SB lesion (L4, upper gastrointestinal location) according to the Montreal classification was determined by MRE and CE as two different diagnostic modalities. The proximal SB lesion (L4) included the jejunum and proximal ileum in this study [23]. Definition of the jejunum and proximal ileum detected by MRE and CE was described earlier. Changes in the Montreal classification by new detection of the proximal lesion (L4) after each examination were analyzed. Findings of the terminal ileum and colon were not included in the results according to the Montreal classification in this study.

### 2.6. Statistical Analysis

Values are presented as mean ± standard deviation for quantitative data and as frequencies (percentages) for categorical data. Chi-square test and Fisher's exact test were performed to evaluate categorical variables. All statistical analyses were performed using SPSS version 20.0 (SPSS Inc., Chicago, IL, USA). The confidence interval (CI) was set at 95% and *P* values of < 0.05 were considered statistically significant.

## 3. Results

### 3.1. Baseline Characteristics

**Figure 1:**
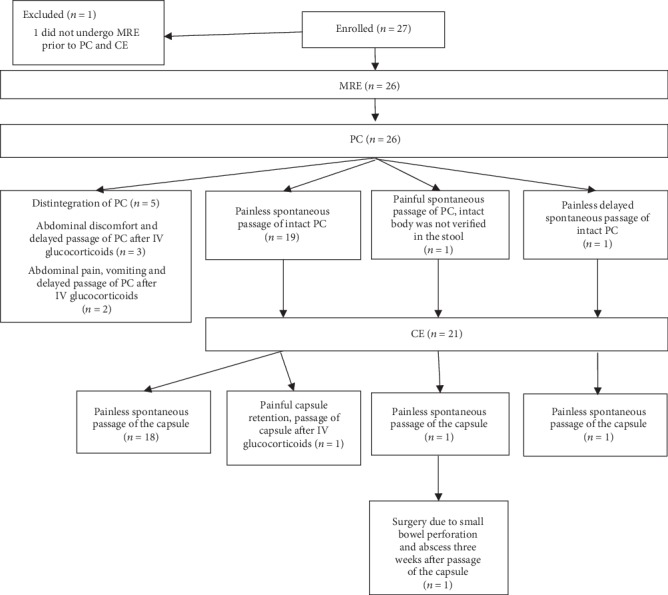
Flow diagram of the study. MRE: magnetic resonance enterography; CE: capsule endoscopy; PC: patency capsule.

A total of 27 patients were enrolled. One patient who did not undergo MRE was excluded. He only underwent CTE, although he underwent PC and CE examinations. Five patients were not eligible for CE due to disintegration of PC. Subsequently, CE was performed for 21 patients. These 21 patients underwent MRE, PC, and CE examinations. The flow diagram of the study is shown in Figure 1. Baseline clinical characteristics of all 26 patients are shown in Table 1. The diagnosis of CD was previously established in 26 patients.

### 3.2. Magnetic Resonance Enterography Findings

MRE detected lesions in 80.8% (21/26) of patients having signs of inflammation with SB wall thickening, hyperenhancement, mural edema, comb sign, enlarged lymph nodes, or presence of ulcers suggesting active CD in SB. No complication related to MRE was observed.

### 3.3. Patency Capsule Findings

PC was performed in 26 patients after MRE. A total of 21 (80.8%) patients who passed the PC intact underwent an actual CE. In 19 patients, PC was not observed on a plain abdominal film at 30 hours after ingestion. They all had no abdominal symptoms related to PC.

**Figure 2:**
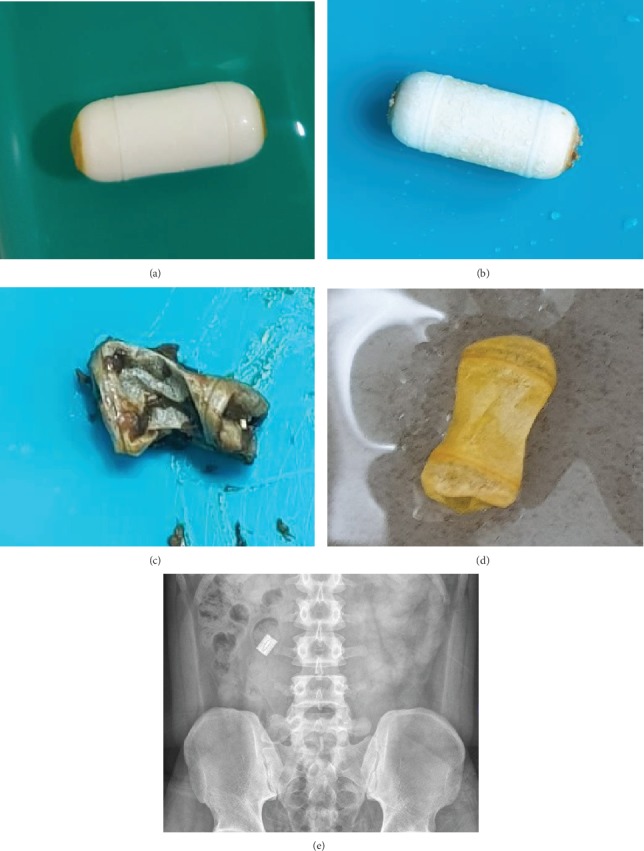
Identification of passed patency capsule: (a, b) intact and (c, d) disintegrated. (e) Patency capsule is observed on an abdominal X-ray image.

Disintegration of the PC was verified by observation of the body of the PC in the stool (Figure 2). It was observed in five patients. They did not undergo an actual CE. Three of these five patients experienced abdominal discomfort during passage of the PC. MRE findings showed SB stricture with 18-20 mm of maximal stricture length. PC was passed without additional management. Disintegrated PC was observed in the stool at three to four days after PC ingestion.

Two of these five patients experienced abdominal pain, nausea, and vomiting after PC ingestion. MRE findings showed SB stricture with 30-110 mm of maximal stricture length. Intravenous glucocorticoids were administered and the disintegrated PC was observed in the stool at five to six days after PC ingestion.

The two remaining patients (a 23-year-old man and a 29-year-old man) out of 26 were described in Discussion.

### 3.4. Capsule Endoscopy Findings

CE was performed in 21 patients after MRE and PC examinations. All patients could properly swallow the capsule. In 18 patients, the capsule was excreted spontaneously without abdominal symptoms. In two of these 18 patients, the capsule did not pass through the ileocecal valve during its working time. The capsule was excreted spontaneously without abdominal symptoms despite a delayed capsule excretion.

However, in a 39-year-old man, capsule retention with abdominal pain occurred in the terminal ileum. Intravenous glucocorticoids were administered and the capsule passed after five days. MRE findings showed wall thickening of the terminal ileum with 20 mm of stricture length. CE findings showed multiple erosions and ulcers in the proximal to the terminal ileum with capsule retention in the terminal ileum.

Regarding the quality of CE images, the overall examination was considered excellent in 14.3% (3/21) of cases and good in 85.7% (18/21) of cases. SB lesions were detected by CE in 81.0% (17/21) of cases.

### 3.5. Comparison of Magnetic Resonance Enterography and Capsule Endoscopy Findings

When the detection rate of lesions by each modality was compared, overall active SB lesions of CD were detected in 76.2% by MRE and 81.0% (*P* = 0.707) by CE in 21 patients. When lesions were analyzed at the segment level (Table 2), the jejunal lesion was detected in 0% (0/21) by MRE and in 23.8% (5/21) (*P* = 0.048) by CE. Two patients had isolated jejunal lesion with erosion on CE which was not detected on MRE. The proximal ileal lesion was detected in 23.8% (5/21) by MRE and in 52.4% (11/21) (*P* = 0.057) by CE. Terminal ileal lesion was detected in 76.2% (16/21) by MRE and in 66.7% (14/21) (*P* = 0.495) by CE.

On the other hand, disintegration of the patency capsule was shown in 5 patients out of 26 patients, who did not undergo CE. When these 5 patients were also included and accounted as negative CE findings, overall active SB lesions of CD were detected in 80.8% by MRE and 65.4% (*P* = 0.211) by CE in 26 patients. When lesions were analyzed at the segment level, the jejunal lesion was detected in 0% (0/26) by MRE and in 19.2% (5/26) (*P* = 0.051) by CE. The proximal ileal lesion was detected in 30.8% (8/26) by MRE and in 42.3% (11/26) (*P* = 0.388) by CE. Terminal ileal lesion was detected in 80.8% (21/26) by MRE and in 53.8% (14/26) (*P* = 0.039) by CE.

**Figure 3:**
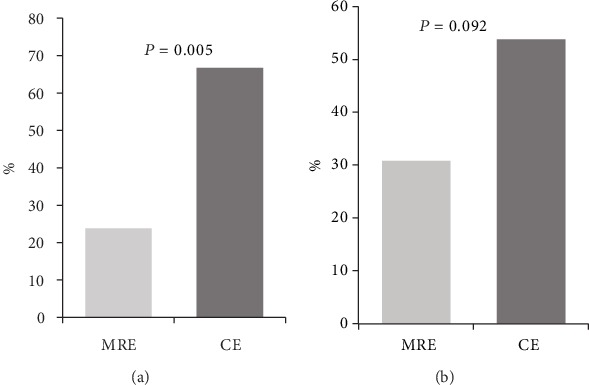
Detection rates of proximal small bowel lesion (L4) according to Montreal classification with magnetic resonance enterography (MRE) and capsule endoscopy (CE). (a) Both MRE and CE were performed in 21 patients (*n* = 21). MRE and CE independently detected proximal disease in 23.8% (5/21) and 66.7% (14/21) (*P* = 0.005), respectively. (b) Disintegration of the patency capsule was shown in 5 patients out of 26 patients. These 5 patients did not undergo CE. These 5 patients were accounted as negative CE findings. All 26 patients underwent MRE (*n* = 26). MRE and CE independently detected proximal disease in 30.8% (8/26) and 53.8% (14/26) (*P* = 0.092), respectively.

Regarding the detection of the proximal SB lesion (L4) according to the Montreal classification (Figure 3), MRE and CE independently detected proximal disease in 23.8% (5/21) and 66.7% (14/21) (*P* = 0.005), respectively.

On the other hand, disintegration of the patency capsule was shown in 5 patients out of 26 patients, who did not undergo CE. When these 5 patients were also included and accounted as negative CE findings, MRE and CE independently detected proximal disease in 30.8% (8/26) and 53.8% (14/26) (*P* = 0.092), respectively.

## 4. Discussion

This study compared diagnostic yields of MRE and CE for the assessment of SB involvement in patients with CD. Results of this study showed that the overall detection rate by CE was similar to that by MRE. However, when lesions were analyzed at a segment level of SB, the detection rate for SB lesions in the jejunum by CE was significantly higher than that by MRE (23.8% vs. 0%, *P* = 0.048) in 21 patiens. Moreover, according to the Montreal classification, CE showed a significantly higher detection rate for proximal SB lesions (L4) than MRE (66.7% vs. 23.8%, *P* = 0.005) in 21 patients. These jejunal and proximal SB lesions which were negative findings on MRE showed relatively mild inflammation on CE findings although some patients had mild strictures. However, when the patients who did not undergo CE due to the disintegration of PC were included in the analysis, the detection rate for proximal SB lesions in 26 patients did not show the statistical differences between MRE and CE.

CE lacks the ability to visualize extraintestinal lesions while MRE could provide information on extraluminal manifestations [24, 25]. In our study, there was no enteric fistula or abscess detected by MRE.

A recent study has reported diagnostic yields of SB lesions by MRE and CE in patients with CD [15]. SB lesions were found in 44.7% (21/47) of cases by MRE and in 76.6% (36/47) (*P* = 0.001) of cases by CE. When analyzing lesions at a segment level of SB, detection rates for SB lesions in all three segments (the jejunum, proximal ileum, and terminal ileum) by CE were significantly higher than those by MRE (*P* = 0.01, *P* = 0.01, and *P* = 0.005, respectively). In our study, CE showed a higher detection rate for the jejunal lesion than MRE among the patients without severe stricture causing capsule retention, although detection rate of overall SB mucosa was similar between CE and MRE.

Another previous study has published the detection rate of proximal SB lesions (L4) according to the Montreal classification by MRE and CE findings [26]. MRE and CE independently detected proximal SB lesions in 26% (20/79) and 51% (29/56) (*P* < 0.01) of patients, respectively. Similarly, in our study, CE showed a higher detection rate for lesions at the proximal SB location (L4) than MRE. This relatively higher detection of lesions at the jejunum or proximal SB location might influence the management of CD, leading to an earlier introduction of an immunomodulator and/or biologics [15, 27].

In our study, MRE detected lesions with mucosal thickening in the proximal ileum that was not detected on CE in three patients. These findings suggest that CE does not always show a higher yield in some patients to detect the proximal ileum than MRE. However, lesions of the terminal ileum in these three patients were all detected by both MRE and CE.

In our study, all 26 patients underwent PC examinations before CE to evaluate the safety and feasibility of PC, although MRE did not show any abnormalities in the SB in some patients (*n* = 5). In 20 patients, PC was passed intact without pain, although MRE findings showed SB strictures in some patients. Painless passages of intact PC were found at 30 hours after PC ingestion in 19 patients. In a 23-year-old man out of 20 patients, the PC was passed intact painlessly in the stool at 48 hours after PC ingestion, not after 30 hours. MRE findings showed multiple mild enlarged lymph nodes along the ileocolic vessels without abnormal SB wall thickening. Subsequent actual CE was also passed spontaneously without any symptoms. Similarly, a previous study on the PC showed painless passage of an intact PC, indicating the safety of CE [28].

In our study, a 29-year-old man experienced abdominal pain during passage of the PC. MRE finding showed multifocal wall thickening of the ileum. Intact body of PC was not verified in the stool. Capsule endoscopic findings showed erosion, ulcer, mucosal erythema, edema, and delayed passage of capsule were noted at the ileum. Painless spontaneous passage of the capsule was verified. However, he underwent surgery because of SB perforation and abscess three weeks after the passage of the actual capsule, although the surgery was not directly associated with PC or CE examination.

In our study, five patients with SB stricture on MRE experienced abdominal discomfort or pain after PC ingestion. Disintegration of the PC was identified, and actual CE was not performed. As previously reported, disintegration of PC or painful passage might be associated with a clinically relevant SB stricture and a high probability of surgery. Thus, CE cannot be performed [28]. This approach for SB patency with PC might have decreased the rate of capsule retention in our clinical practice. The present study showed all patients with 20 mm or longer length of stricture on MRE had passages of disintegrated PC or capsule retention. Therefore, 20 mm or longer length of SB stricture on MRE may predict capsule retention. Thus, in these patients, CE is not helpful. Similarly, a previous study published that an average 97 mm stricture length on MRE was found to be the predictive features for PC retention [1].

Capsule retention can occur in up to 13% of patients with established CD and in 1.6% of patients with suspected CD [29]. MRE can help predict SB stricture and capsule retention. However, MRE can underestimate SB stricture in mild diseases or overestimate it in more severe diseases [1, 15]. Therefore, a previous study has suggested that patients with positive prediction of capsule retention based on MRE findings should undergo PC before the actual CE [1]. However, there might be false-negative PC examination for capsule retention. In a 39-year-old man in our study, although the PC was passed intact spontaneously without abdominal symptom, capsule retention with abdominal pain occurred in the terminal ileum. Another 28-year-old man who did not undergo the MRE (and underwent only CTE) was excluded for the final statistical analysis. He showed spontaneous painless PC passage and asymptomatic capsule retention in the distal ileum. The capsule passed spontaneously after 17 days. The CTE findings before PC examination were wall thickening and increased mural enhancement of the distal ileum.

As the SB is involved in over 70% of CD patients, its evaluation is useful for initial diagnosis and assessment of mucosal inflammation, which is important for the management of patients [13, 30]. MRE and CE have comparable diagnostic accuracies. However, CE is more sensitive for the detection of subtle mucosal inflammation and proximal SB involvement [13, 30], similar to the results of our study. A previous prospective study on changes over time with regard to the location and behavior of CD showed that the risk of developing stricturing or penetrating complications existed even when the disease in the SB was clinically quiescent [26]. In patients with additional segments of active disease identified in the SB, intensive monitoring may potentially lead to the earlier detection of disease features associated with a more severe prognosis [26].

This study has a few limitations. First, there is no gold standard of diagnostic modality for the detection of SB lesions. And SB enteroscopy was not performed to identify SB lesions in this study. In a previous study on SB balloon enteroscopy in patients with CD, the enteroscope reached the jejunum in 40% of cases and the proximal ileum in 98% of cases [31]. Enteroscopy provides direct visualization of SB mucosa and enables pathologic diagnosis by forcep biopsy. However, enteroscopy is less sensitive for detecting proximal SB lesions because it frequently could not reach the entire SB. Second, MRE activity was not stratified according to the inflammatory severity and was not classified into specific degrees using previous alleged scoring systems such as magnetic resonance index of activity (MaRIA), magnetic resonance enterography global score (MREGS), or Crohn's disease activity score (CDAS) [32]. Third, the degree of inflammation on CE was not stratified according to the inflammatory severity and was not quantified using a previous scoring index such as the Lewis score [33]. Fourth, diffusion-weighted imaging MRE (DWI-MRE) was not analyzed in detail although it was taken into consideration for imaging interpretation. It was because a recent meta-analysis of 1066 bowel segments for assessment of inflammatory severity concluded that DWI-MRE accuracy was very heterogeneous between studies and was likely overestimated in some studies [34]. Fifth, the detection of the SB lesion by MRE and CE was interpreted by a different reader from a different institution. However, these limitations represent the real-life nature of the present study.

Disintegration of the patency capsule was shown in 5 patients out of 26 patients, who did not undergo CE. In conclusion, when these 5 patients were accounted as negative CE findings, the diagnostic yields of MRE and CE for the assessment of SB CD including proximal SB lesions were similar. MRE is a more objective tool for detecting clinically relevant stricture than PC although PC examination could be performed safely before the CE to prove the patency of SB. If the PC disintegrates or causes pain during its passage, it indicates a relevant SB stricture. Thus, CE cannot be performed.

## Figures and Tables

**Table 1:** Baseline clinical characteristics of patients with established Crohn's disease (*n* = 26).

Age (y)	38.7 ± 12.8
Men	19/26 (73.1)
Previous abdominal surgery	1/26 (3.8)
Patency capsule examination	26/26 (100)
Actual capsule endoscopy	21/26 (80.8)
White blood cells (×10^3^/*μ*L)	6872.4 ± 2609.2
C-reactive protein (mg/dL)	0.5 ± 1.5

Values are presented as *n* (%) or mean ± standard deviation.

**Table 2:** Diagnostic yields of magnetic resonance enterography (MRE) and capsule endoscopy (CE) according to segments of small bowel lesions. These 5 patients did not undergo CE. These 5 patients were accounted as negative CE findings. All 26 patients underwent MRE (*n* = 26).

		A (*n* = 21)			B (*n* = 26)	
Small bowel lesions	MRE, *n* (%)	CE, *n* (%)	*P* value	MRE, *n* (%)	CE, *n* (%)	*P* value
Jejunum	0 (0)	5 (23.8)	0.048	0 (0)	5 (19.2)	0.051
Proximal ileum	5 (23.8)	11 (52.4)	0.057	8 (30.8)	11 (42.3)	0.388
Terminal ileum	16 (76.2)	14 (66.7)	0.495	21 (80.8)	14 (53.8)	0.039

A: both MRE and CE were performed in 21 patients (*n* = 21). B: disintegration of patency capsule was shown in 5 patients out of 26 patients.

## Data Availability

The clinical data used to support the findings of this study are restricted by the institutional review board of the research ethics committee of Dongguk University Ilsan Hospital in order to protect the patients' privacy.
